# A novel dataset of potato leaf disease in uncontrolled environment

**DOI:** 10.1016/j.dib.2023.109955

**Published:** 2023-12-12

**Authors:** Nabila Husna Shabrina, Siwi Indarti, Rina Maharani, Dinar Ajeng Kristiyanti, Irmawati, Niki Prastomo, Tika Adilah M

**Affiliations:** a Department of Computer Engineering, Universitas Multimedia Nusantara, Tangerang 15111, Banten, Indonesia; b Department of Plant Protection, Universitas Gadjah Mada, Sleman 55281, Yogyakarta, Indonesia; c Department of Information System, Universitas Multimedia Nusantara, Tangerang 15111, Banten, Indonesia; d Department of Physics Engineering, Universitas Multimedia Nusantara, Tangerang 15111, Banten, Indonesia; e Department of Information Technology, Universitas Bina Sarana Informatika, Jakarta Pusat 10450, DKI Jakarta, Indonesia

**Keywords:** Dataset, Image classification, Potato leaf disease, Precision agriculture, Uncontrolled environment

## Abstract

Potatoes are of the utmost importance for both food processing and daily consumption; however, they are also prone to pests and diseases, which can cause significant economic losses. To address this issue, the implementation of image processing and computer vision methods in conjunction with machine learning and deep learning techniques can serve as an alternative approach for quickly identifying diseases in potato leaves. Several studies have demonstrated promising results. However, the current research is limited by the use of a single dataset, the PlantVillage dataset, which may not accurately represent the diverse conditions of potato pests and diseases in real-world settings. Therefore, a new dataset that accurately depicts various types of diseases is crucial. We propose a novel dataset that offers several advantages over previous datasets, including data obtained in an uncontrolled environment that results in a diverse range of variables such as background and image angles. The proposed dataset comprises 3076 images categorized into seven classes, including leaves attacked by viruses, bacteria, fungi, pests, nematodes, phytophthora, and healthy leaves. This dataset aims to provide a more accurate representation of potato leaf diseases and facilitate advancements in the current research on potato leaf disease identification.

Specifications Table

**Table:** 

Subject	Computer Science, Agricultural Science
Specific subject area	Computer vision, Image classification, Deep Learning, Machine Learning, Precision Agriculture
Data format	Raw
Type of data	Image
Data collection	Dataset were obtained at two distinct periods: August 2, 2023, for potato farms located in Magelang, Central Java, Indonesia and August 15-16, 2023, for potato farms located in Wonosobo, Central Java, Indonesia. Images were captured by several team members using various smartphone cameras. The experts from the Department of Plant Protection were then conducted a thorough evaluation of each image and categorized them into seven distinct classes, including “virus,” “phytophthora,” “nematode,” “fungi,” “bacteria,” “pest,” and “healthy.” All images were then resized to 1500 × 1500 pixels resulting in uniform resolution for all images. Images were saved into .jpg format for compatibility with various image-processing software packages, and ease of accessibility
Data source location	The dataset was obtained in Central Java, Indonesia, with the detail location as follows. 1.-7.231694, 109.937778 Tieng, Kejajar, Wonosobo 2.-7.231114, 109.937078 Tieng, Kejajar, Wonosobo 3.-7.205085, 109.911120 Dieng, Kejajar, Wonosobo 4.-7.446640, 110.378579 Kragilan, Pakis, Magelang 5.-7.439587, 110.397223 Kenalan, Pakis, Magelang 6.-7.211900, 109.928986 Parikesit, Kejajar, Wonosobo 7.-7.210166, 109.909267 Dieng Kulon, Batur, Banjarnegara 8.-7.210113, 109.909820 Dieng Kulon, Batur, Banjarnegara 9.-7.198797, 109.915172 Dieng, Kejajar, Wonosobo 10.-7.236805, 109.941532 Tieng, Kejajar, Wonosobo 11.-7.441006, 110.371796 Kaponan, Pakis, Magelang 12.-7.407529, 110.381297 Sumberejo, Ngablak, Magelang
Data accessibility	Repository name: The datasets are publicly and freely available on Mendeley data repository. Data identification number: 10.17632/ptz377bwb8.1 Direct URL to data: https://data.mendeley.com/datasets/ptz377bwb8/1

## 1 Value of the Data

• This dataset was collected in an uncontrolled environment, resulting in a variety of variables, including the background and the diverse directions and distances of the images.
• This dataset is better at representing the various types of diseases commonly found on potato leaves by categorizing them into seven classes, including leaves symptoms attacked by viruses, bacteria, fungi, pests, nematodes, phytophthora, and healthy leaves.
• This dataset can be used for computer vision and pattern recognition, which are primarily employed in classification tasks.
• The potato leaf dataset has motivated researchers to develop a novel approach for classifying potato leaf diseases in uncontrolled environments.
• This dataset will encourage researchers to classify or build models to identify potato leaf pests and diseases using advanced computer vision techniques under background clutter and occlusion conditions.

## 2 Background

The implementation of image processing and computer vision methods can serve as an alternative approach to accelerate the process of identifying diseases in potatoes through the symptoms of leaves. However, the use of a single dataset may impede the ability of Machine Learning (ML) or Deep Learning (DL) to generalize, diversify, and adjust to diverse situations. Furthermore, the existing PlantVillage dataset [1] utilizes a controlled environment for image capture, whereby each image is captured under controlled parameters such as a clean background, controlled angles, and camera directions. This approach was adopted to enable system designers to minimize various sources of variability and achieve high-performance results. In addition, the PlantVillage dataset comprises only three classes: healthy, early blight, and late blight. Among these, late and early blight are caused by fungi. Although previous studies have demonstrated superior performance, the available datasets may not accurately represent the real-world conditions of potato pests and diseases because of the controlled environment in which the images were captured and the lack of information on disease type, which only captures diseases caused by fungi. The creation of a dataset specifically for diversifying images of potato leaf diseases in uncontrolled environments is highly needed because of the scarcity of suitable image datasets for a wider range of diseases. To address this issue, we have recently acquired novel primary data that offer several advantages over previous datasets and will better represent the various types of diseases commonly found on potato leaves.

## 3 Data Description

The dataset was developed by multidisciplinary teams from the Faculty of Engineering and Informatics, Universitas Multimedia Nusantara, and Faculty of Agriculture, Universitas Gadjah Mada. The dataset was collected from several potato farms in Java Island, Indonesia, primarily in central Java. The dataset was gathered in an unrestricted setting characterized by multiple inconsistencies, such as background and diverse directions and distances. This dataset comprises several categories of potato leaf diseases, including those caused by fungi, viruses, pests, bacteria, phytoplasmas, and nematodes.

**Fig. 1:**
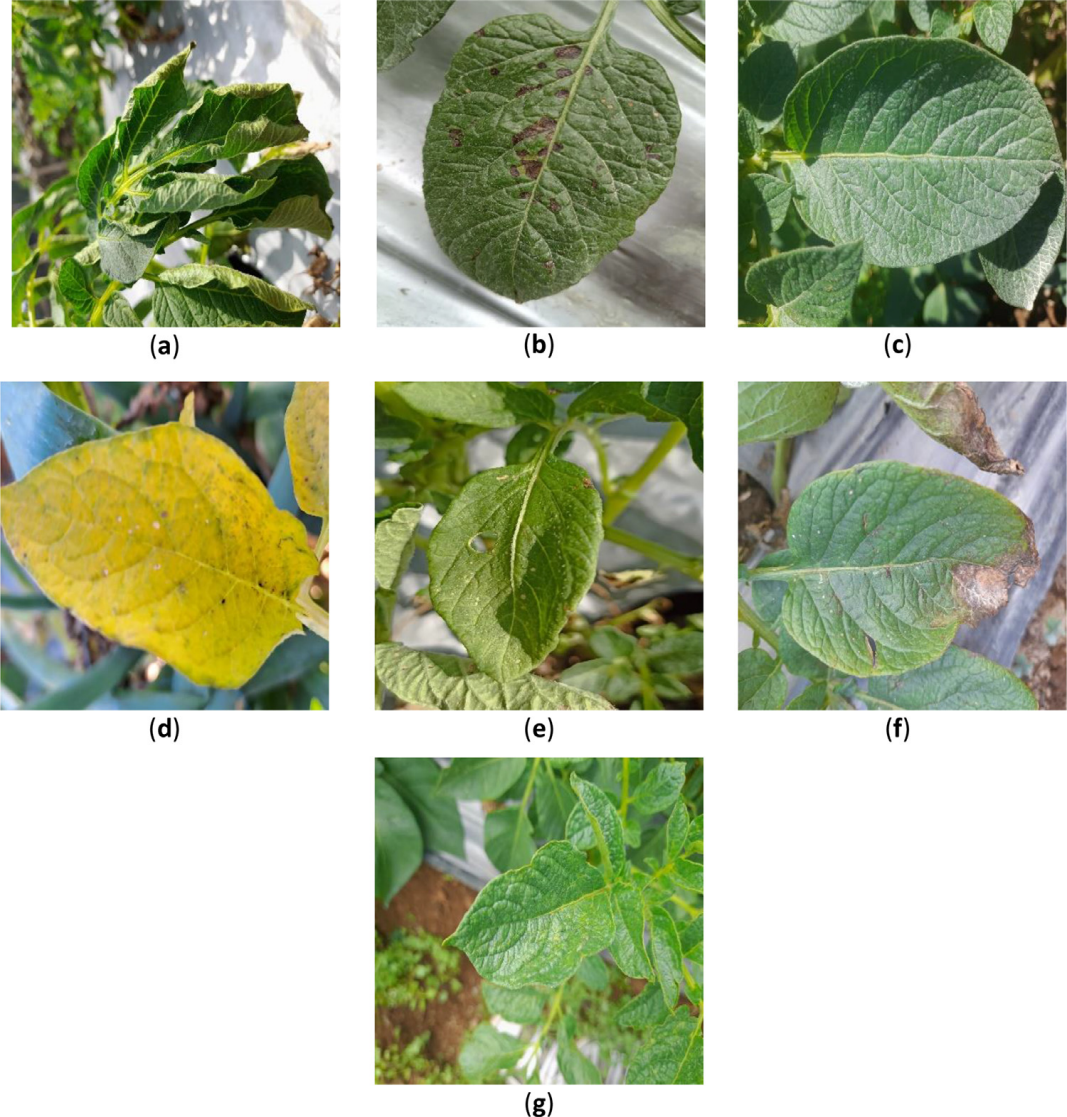
Sample of the seven categories of potato leaf dataset: (a) bacteria; (b) fungi; (c) healthy; (d) nematode; (e) pest; (f) phytophthora; (g) virus.

The sample images for each category are shown in Fig. 1. The total number of images per category in the dataset is listed in Table 1. As listed in Table 1, the dataset consists of seven classes, with a total of 3076 images. The classes were categorized as “virus,” “phytophthora,” “nematode,” “fungi,” “bacteria,” “pest,” and “healthy”. The images had a resolution of 1500 × 1500 pixels and were saved in. jpg format to ensure ease of access and compatibility with various image-processing software packages.

**Table 1:** Distribution of the dataset.

Class	Number of images
Virus	532
Phytophthora	347
Nematode	68
Fungi	748
Bacteria	569
Pest	611
Healthy	201
**Total**	**3076**

## 4 Experimental Design, Materials and Methods

### 4.1 Image capturing

The images used in this study were obtained from multiple angles and distances ranging from approximately 5–15 cm. The pictures were acquired under diverse weather conditions, including sunny, cloudy, and partially cloudy. Images were captured between the hours of 8 a.m. and 3:00 p.m This dataset collected symptoms of pathogen and pest attacks on potato leaves of varying ages, approximately 35-80 days after planting. At this stage, the symptoms caused by each pathogen that appeared in the leaves are visible and have no ambiguities with other infections. However, because the dataset was intended for the image classification task, the disease progression stage was not included in the dataset. The disease stage was randomly selected based on the occurrence of diseases observed in the field. The images were obtained at two distinct periods: August 2, 2023, for potato farms located in Magelang, Central Java and August 15–16, 2023 for potato farms located in Wonosobo, Central Java. The locations of potato farms are listed in Table 2.

**Table 2:** Location of potato farm.

Coordinate	Location	Meter above the sea (MASL)
−7.231694, 109.937778	Tieng, Kejajar, Wonosobo	±1802
−7.231114, 109.937078	Tieng, Kejajar, Wonosobo	±1807
−7.205085, 109.911120	Dieng, Kejajar, Wonosobo	±2064
−7.446640, 110.378579	Kragilan, Pakis, Magelang	±1208
−7.439587, 110.397223	Kenalan, Pakis, Magelang	±1428
−7.211900, 109.928986	Parikesit, Kejajar, Wonosobo	±1992
−7.210166, 109.909267	Dieng Kulon, Batur, Banjarnegara	±2062
−7.210113, 109.909820	Dieng Kulon, Batur, Banjarnegara	±2061
−7.198797, 109.915172	Dieng, Kejajar, Wonosobo	±2128
−7.236805, 109.941532	Tieng, Kejajar, Wonosobo	±1642
−7.441006, 110.371796	Kaponan, Pakis, Magelang	±1155
−7.407529, 110.381297	Sumberejo, Ngablak, Magelang	±1281

**Fig. 2:**
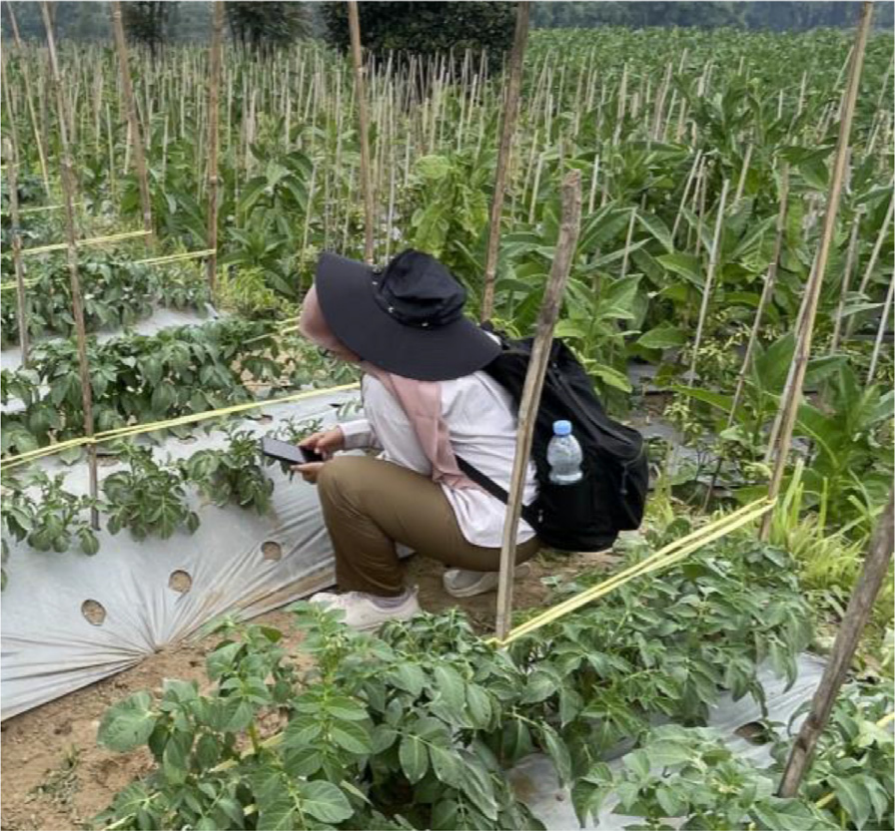
Process of capturing images by team members.

Images were captured by several team members using various smartphone cameras. The detailed specifications of the smartphone camera are listed in Table 3. The size format for the captured images had a variety of resolutions ranging from 2448 × 3264 to 3472 × 4624, encompassing both vertical and horizontal forms. This is because of the different specifications of the smartphone cameras used to capture the images. The aspect ratios of the images captured by the smartphone cameras were 4:3 and 16:9. Furthermore, the different positions of the cameras used by the teams resulted in various backgrounds and occlusion of the images. Fig. 2 shows how the team members gathered the images.

**Table 3:** Specifications of smartphone cameras used to capture dataset images.

Smart phone	Camera specifications
iPhone 6	8 MP, f/2.2 aperture
iPhone 7	12 MP, ƒ/1.8 aperture
iPhone 7+	12 MP wide-angle, ƒ/1.8 aperture
iPhone X	12 MP wide-angle, ƒ/1.8 aperture
iPhone 12 mini	12 MP wide angle, ƒ/1.6 aperture
Samsung galaxy a33	48 MP, f/1.8 aperture
Samsung SM A14SF	50 MP main camera, f/1.8 aperture
Samsung M12	48 MP main camera, f/2.0 aperture
Samsung Galaxy A03s	13 MP, f/2.0 aperture
Vivo T1	50 MP, f/1.8 aperture
Redmi Note 7	48 MP (wide), f/1.8 aperture
Xiaomi Redmi Note 10S	48 MP (wide), f/1.79 aperture

### 4.2 Image labelling

The experts from the Department of Plant Protection, Faculty of Agriculture, Universitas Gadjah Mada, who have expertise in plant disease, specifically for potato leave disease, conducted a thorough evaluation of each image and labelled them into seven distinct classes, including “virus,” “phytophthora,” “nematode,” “fungi,” “bacteria,” “pest,” and “healthy.” The labelling process was performed by meticulous observation of the visual characteristics of the captured images, utilizing the core symptoms of each disease. Images exhibiting ambiguous traits such as the concurrent presence of multiple disease features were excluded from the final dataset.

The labelling process utilizes visual observation, performed by experts in plant disease by relying on symptoms for each of the disease categories, such as leaf discoloration, wilting, spots, lesions, or abnormal growth patterns, which can provide clues about the nature of the disease. Reference materials and disease manuals were also used. In addition, plant disease experts labelled the images based on field observations. They considered factors such as weather conditions, plant history, and common diseases in the area. The combination of field observations, experience, and visual inspection of the symptoms appearing in the leaves resulted in the final disease category. A summary of each visual characteristic of the potato leaf dataset is provided in Table 4.

**Table 4:** Summary of visual characteristics of potato leaves.

Class	Characteristic
Virus	reduced leaf size and crinkling, mild mottling or mosaic, necrosis
Phytophthora	leaves observed as dark brown to black lesions, can cause the lesions to enlarge into circular and necrotic patches
Nematode	have yellowish leaves with symptoms similar to those of water and nutrient deficiencies
Fungi	circular patterns manifested along leaf edges and/or slightly sunken leaf spot with yellow borders and concentric ring appearance, and/or yellow leaves with powdery patches
Bacteria	symptoms on leaves wilt without dead or necrotic leaves; the leaves plant initially does not turn yellow
Pest	leaf tissue became distorted and/or with holes and/or dotted leaves with silver colour or chlorotic material and/or with mined route on leaves
Healthy	uniform green colour in all parts of the leaves and perfect leaf shape without imperfections

*Phytophthora infestans* symptoms on leaves can be observed as dark brown to black lesions, and if left untreated, they can cause lesions to enlarge into circular and necrotic patches [2]. The symptoms examined in this investigation encompassed dark black lesions that covered the majority of the leaves, ranging from 20% to 90% of the surface area, which eventually dried out, did not sporulate, and exhibited a tan hue. Certain photographs depicted infections in the form of small lesions, ranging in color from bright green to dark green, exhibiting circular to irregular shapes, and displaying wet spots, as described by [3].

Fungal diseases in potato leaves can show different symptoms, depending on the causative organism. Early blight caused by *Alternaria solani* on leaves can be recognized as circular patterns manifested along leaf edges [4], slightly sunken leaf spots with yellow borders, and concentric rings. In certain cases, these spots may converge. This characteristic becomes more pronounced as the pathogen infects the underside of the leaf, displaying light-brown spots [5]. However, it should be noted that this development does not lead to leaf drying, as previously described by [6]. Another fungal disease is characterized by yellow leaves that become necrotic with powdery patches [2].

Bacteria-caused diseases show symptoms on leaves as secondary symptoms because of infection of tubers and stems. Symptoms on leaves wilt without dead or necrotic leaves; wilt is rapid and the plant does not initially turn yellow. When an infected lower stem is placed in water, a prominent milky ooze is observed [2]. This symptom attack may or may not be visible depending on the development of the disease and its relationship with temperature. Wilt symptoms occur when vascular infection occurs, which inhibits nutrition in the stem and leaf petioles [7]. In this study, we did not use leaves with symptoms of primary bacterial attack.

Nematode infections above the ground can be seen as expanding patches with poor growth in the field. The plants are smaller and have yellowish leaves, with symptoms similar to those of water and nutrient deficiency. Plants with damaged roots become wilted, particularly under warmer temperatures during the day, and may remain wilted even with irrigation [8].

Viral symptoms include reduced leaf size and crinkling, mild mottling or mosaicism, necrosis, and severe infections that can cause dwarfing in plants. In addition to these diseases, crop losses in potatoes worldwide can be caused by pests. Potato pests can be broadly grouped into three categories: sucking pests, tuber- and root-damaging pests, and foliage feeders or defoliating pests [9]. Damage from thrips can be observed in leaf tissues, which become distorted and dotted with silver or chlorotic material. When the leaves are fed continuously, their tips wither, coil up, and eventually die more severely under dry weather conditions. Both nymphs and adults graze on leaves along the midribs and veins. Foliage feeders, caterpillars, leaf beetles, and grasshoppers feed on leaves by scraping and skeletonizing them. Older larval instars are divided into groups that feed heavily on frequently defoliating leaves. If the infection is severe, the crop can simultaneously lose its leaves [9]. Another pest is the leaf miner insect, *Liriomyza spp.*, which causes leaves to be mined by their larvae [10].

### 4.3 Image pre-processing

**Fig. 3:**
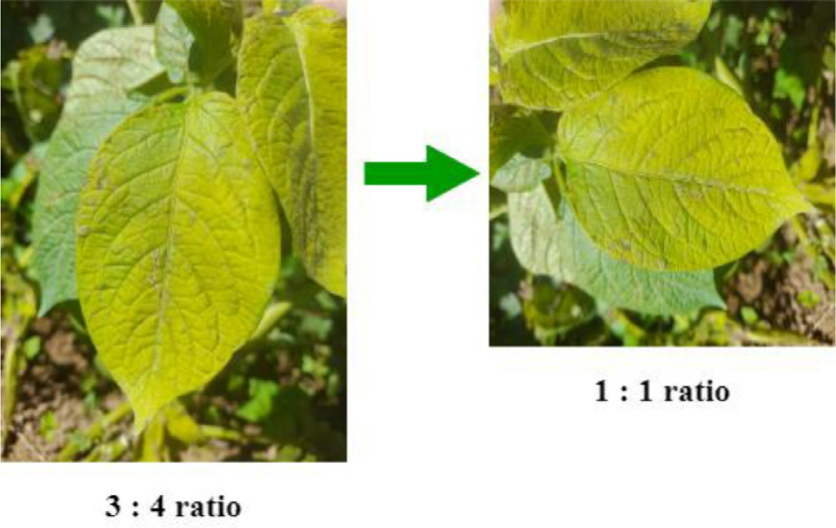
Preprocessing stage for resizing the original captured image in a 1:1 ratio.

The images were captured at 4:3 or 16:9 ratios in the horizontal direction and 3:4 or 9:16 ratios in the vertical direction. The native resolution of the Convolutional Neural Network (CNN)-based method was 1:1 aspect ratio. Therefore, the gathered images were resized to a 1:1 ratio by using a script in Python with the Pillow Library to match most of the CNN inputs. All images were then resized to 1500 × 1500 pixels using the same script, resulting in uniform resolution for all images. Images were saved into .jpg format for compatibility with various image-processing software packages, and ease of accessibility. The resizing process is illustrated in Fig. 3.

### 4.4 Comparison with existing dataset

This section provides a comparison and novelty of the proposed dataset with the existing PlantVillage dataset. Table 5 provides a comparison between the PlantVillage and proposed datasets.

**Table 5:** Comparison between PlantVillage datasets and our proposed dataset.

Comparison	PlantVillage dataset	Our proposed dataset
Class	Three categories: early blight (fungi), late blight (fungi-like), and healthy	Seven categories: virus, phytophthora, nematode, fungi, bacteria, pest, and healthy
Total images	2052 images	3076 images
Environment	Controlled environment: • clear and uniform background • uniform angle of view • uniform object placement and orientation • uniform lighting condition	Uncontrolled environment: • varied background and occlusion • varied angle of view • diverse object placement and orientation • varied lighting condition
Dimension (in pixel)	256×256 (low resolution)	1500×1500 (high resolution)

**Fig. 4:**
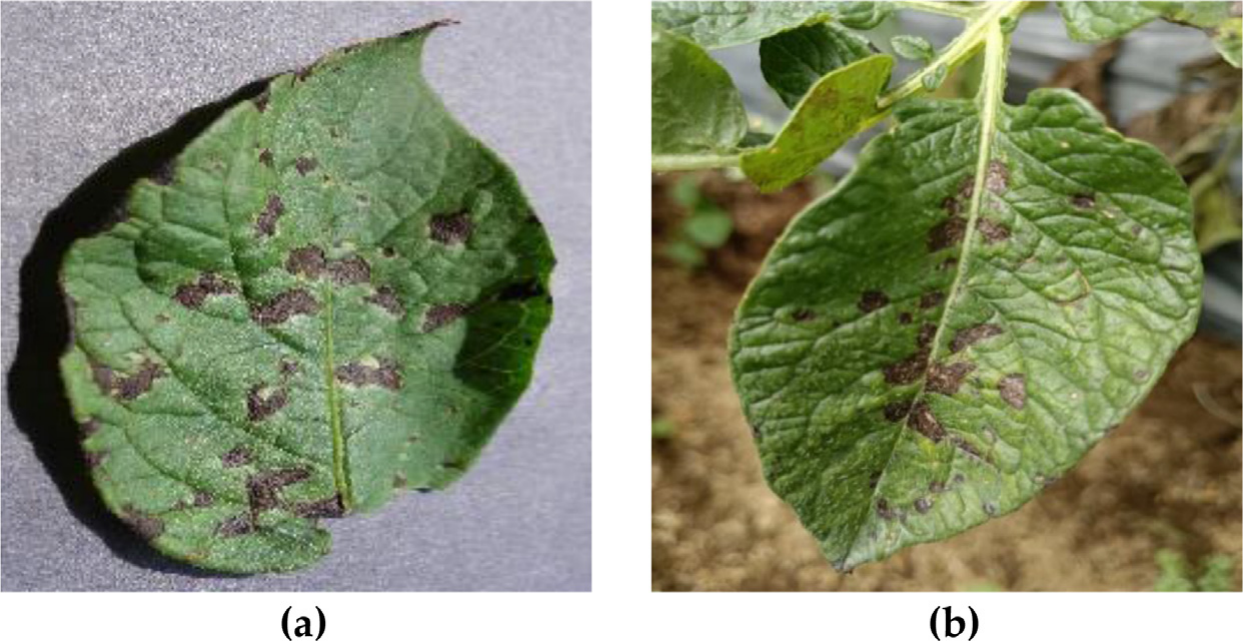
The sample of potato leaves in fungi category in (a) the PlantVillage dataset, and (b) the proposed dataset.

**Fig. 5:**
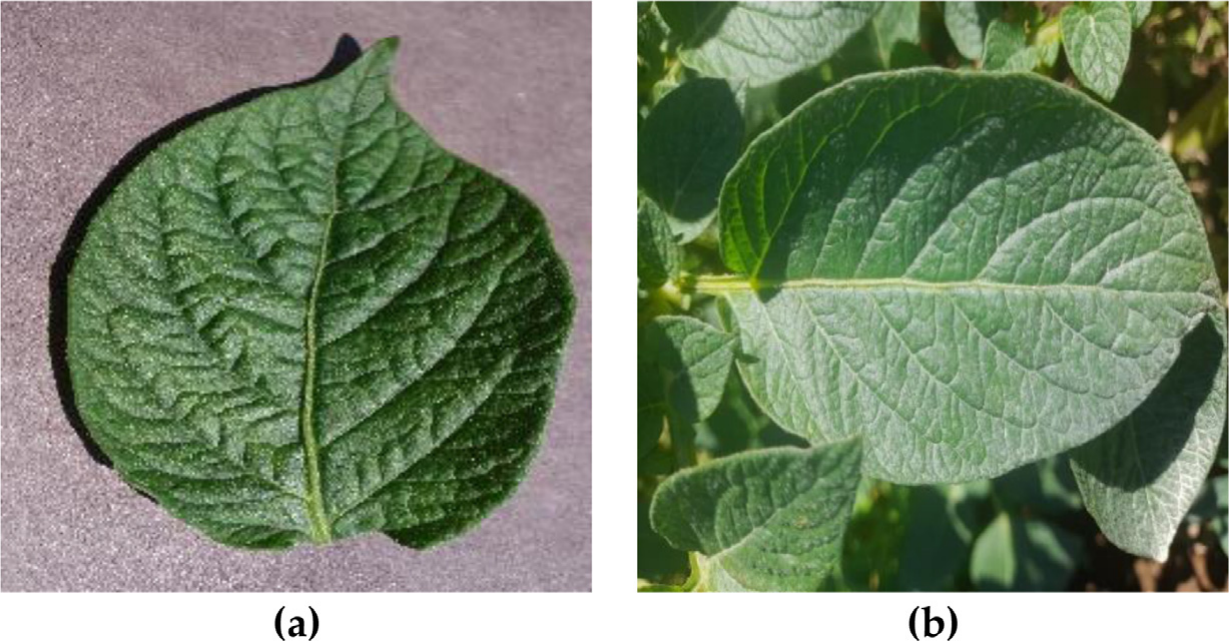
The sample of potato leaves in the healthy category in (a) the PlantVillage dataset, and (b) the proposed dataset.

As presented in Table 5, the PlantVillage dataset includes only images of leaf symptoms caused by fungi. In contrast, our dataset included images of leaf symptoms caused by a variety of sources, including viruses, phytophthora, nematodes, fungi, bacteria, and pests. The PlantVillage dataset was collected in a controlled environment with uniform background, angle, orientation, and lighting conditions. Additionally, the images were captured at a low resolution with a pixel size of 256 × 256. The proposed dataset addresses the shortcomings of the PlantVillage dataset by presenting images in a broader range of symptom categories, capturing uncontrolled environments, and boasting a higher resolution. Fig. 4 shows samples of the images from the PlantVillage dataset and our proposed dataset from the fungi category, and Fig. 5 shows samples from the healthy class.

Although the PlantVillage dataset offers benchmark information and training materials for testing and evaluating algorithms for the classification of potato leaf diseases, it may exhibit limitations in encompassing a broader range of potato leaf diseases. Consequently, the model trained on this dataset can encounter constraints when dealing with unfamiliar categories or various real-world situations. This difference signifies that our proposed dataset provides diverse conditions for achieving a robust model performance in real-world settings.

### 4.5 Preliminary study

#### 4.5.1 Proposed preliminary study

After collecting the dataset, we conducted a preliminary study using several pre-trained CNN: EfficientNetV2B3 [11,12], MobileNetV3-Large [13], VGG-16 [14], ResNet50 [15], and DenseNet121 [16]. This CNN-based model was selected because of its lightweight size and outstanding performance on the ImageNet dataset. In addition, previous research has shown that these family models perform well for leaf disease classification using the PlantVillage dataset [17,18].

The dataset was first split into training and test datasets in a ratio of 90:10, consisting of 2765 and 311 images for training and testing, respectively. Before training the CNN modes, the training set was split into 2489 for training and 276 images for validation. All images were then resized to 224 × 224 pixels to match the input specifications of all tested CNN models. The preliminary study was conducted on the Google Colab Free version utilizing the Keras and TensorFlow Library. All CNN models were trained using pre-trained models with pretrained weights on the ImageNet dataset. For consistency, all models were trained using the Adam optimizer with a learning rate of 0.0001, a categorical cross-entropy loss function, and batch sizes of 64 and 50 epochs.

**Fig. 6:**
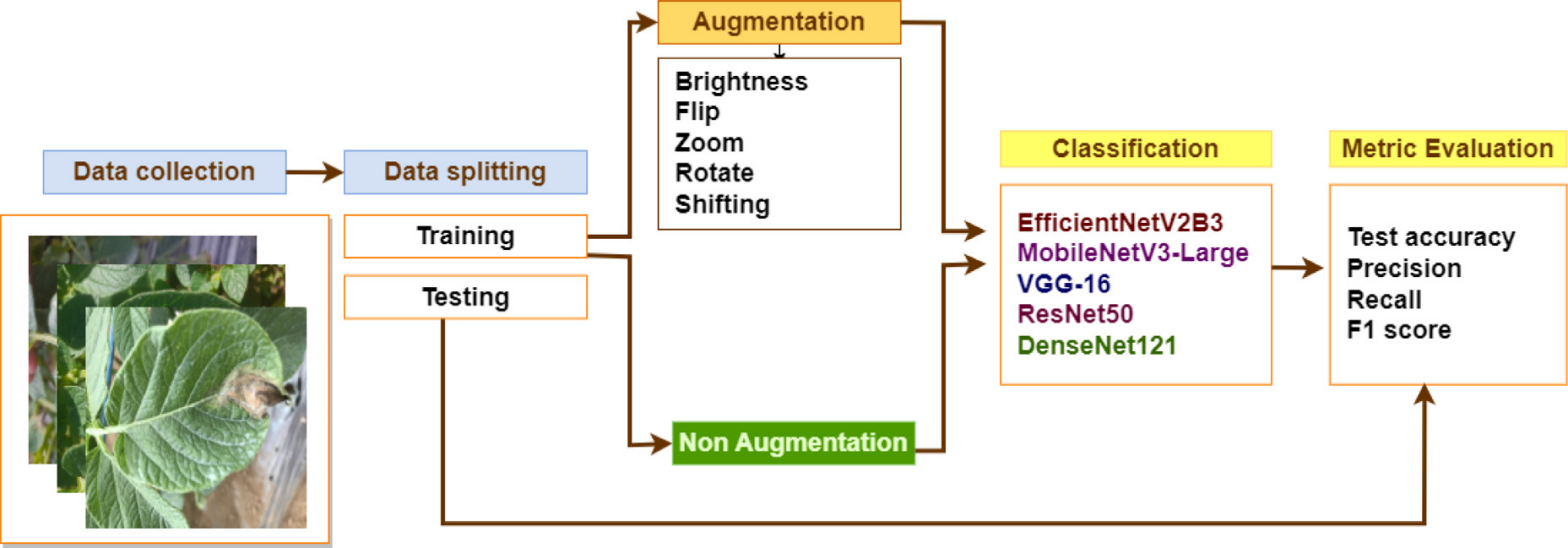
Experimental study using the proposed dataset.

A schematic of the experimental setup is shown in Fig. 6. Two scenarios were employed in this study: training without data augmentation, and training with data augmentation. The second scenario was conducted by adding additional preprocessing steps using data augmentation, including brightness, flipping both horizontal and vertical, rotating, zooming, and shifting both horizontal and vertical, which resulting of around 990 up to 1000 images per class. Data augmentation was utilized owing to the imbalance issue in the dataset. Several evaluation metrics were employed in this study, such as the test accuracy, precision, recall, and F1 score. Eqs. (1)–(4) present the formulae for each metric. Most of the evaluation metrics employed TP, FP, TN, and FN, where TP is a True Positive; FP is a False Positive; TN is a True Negative; and FN is a False Negative.

(1) $$Test\;Accuracy=\;\frac{TP+TN}{TP+TN+FP+FN}$$

(2) $$Precision=\;\frac{TP}{TP+FP}$$

(3) $$Recall=\;\frac{TP}{TP+FN}$$

(4) $$F1\;score=\;\frac{2\;x\;Precision\;x\;Recall}{Precision+Recall}$$

#### 4.5.2 Result of the preliminary study

Tables 6 and 7 list the performance results of the tested model for scenarios without and with augmentation, respectively. In the scenario in which the dataset did not undergo augmentation, as presented in Table 6, EfficientNetV2B3 displayed the highest test accuracy of 0.7363, followed by MobileNetV3-Large at 0.7203. In contrast, VGG-16 demonstrated the lowest test accuracy with a value of 0.5981. In the second scenario, in which the dataset was augmented, EfficientNetV2B3 again emerged as the top-performing model, with a test accuracy of 0.723, as shown in Table 7. MobileNetV3-Large followed closely, with a test accuracy of 0.7042, whereas VGG-16 continued to exhibit the poorest performance, with a test accuracy of 0.5627. These results indicate that the model performance remained subpar, even after the addition of augmentation to balance the dataset. This is because of the complexity of our proposed dataset, which encompasses a diverse range of backgrounds and image angles, resulting in an augmentation process that does not yield significant improvements.

**Table 6:** Experimental result from non-augmented dataset.

Model	Test accuracy	Precision	Recall	F1-score
EfficientNetV2B3	0.7363	0.7428	0.7363	0.7302
MobileNetV3-Large	0.7203	0.7316	0.7203	0.7131
VGG-16	0.5981	0.6054	0.5981	0.5904
ResNet50	0.6817	0.7006	0.6817	0.6748
DenseNet121	0.5916	0.6058	0.5916	0.5911

**Table 7:** Experimental result from augmented dataset.

Model	Test accuracy	Precision	Recall	F1-score
EfficientNetV2B3	0.7235	0.7378	0.7235	0.7199
MobileNetV3-Large	0.7042	0.7092	0.7042	0.7037
VGG-16	0.5627	0.5697	0.5627	0.5607
ResNet50	0.6624	0.6659	0.6624	0.6607
DenseNet121	0.5852	0.5858	0.5852	0.5847

Table 8 compares the performance of the best-performing model in this preliminary study, EfficientNetV2B3, when trained on the PlantVillage dataset and our proposed dataset. As shown in Table 8, EfficientNetV2B3 achieved a test accuracy of 98.15% when trained on the PlantVillage dataset. However, when trained on the proposed dataset, the accuracy of the model was 73.63%. These results suggest that while the model performed well on the PlantVillage dataset, it struggled when trained on our proposed dataset. Given the exceptional and distinctive characteristics of our proposed dataset, it is understandable that the model struggled to perform optimally. The lack of a controlled environment within the dataset poses a significant challenge for the model to effectively learn.

**Table 8:** Comparison of results with other datasets.

Model	Dataset	Test accuracy
EfficientNetV2B3	PlantVillage dataset	98.15%
EfficientNetV2B3	**Our dataset**	**73.63%**

The results of the experimental comparison indicate that the EfficientNetV2B3 model, which exhibits exceptional classification performance on the PlantVillage dataset, underperforms when applied to the proposed dataset. Therefore, it is necessary to develop models with improved performance for identifying potato leaf pests and diseases in uncontrolled environments. The real-world scenarios presented in our dataset can aid the algorithm in handling diverse situations, leading to improved recognition accuracy and facilitating optimization and refinement of the algorithm. This will enable researchers to train more advanced algorithms. We hope that the release of this dataset will lead to the development of an automatic potato leaf disease identification system that can be used in real-life scenarios and will contribute to the advancement of precision agriculture.

## Limitations

Adding potato leaf disease samples from countries outside Indonesia could enhance the diversity of the dataset.

## Ethics Statement

The dataset presented in this work does not include tests on animals or humans. All images used were obtained by the authors and do not come from any other source.

## CRediT authorship contribution statement

**Nabila Husna Shabrina:** Conceptualization, Methodology, Software, Formal analysis, Investigation, Resources, Data curation, Writing – original draft, Writing – review & editing, Visualization, Project administration, Funding acquisition. **Siwi Indarti:** Conceptualization, Methodology, Validation, Formal analysis, Investigation, Resources, Data curation, Writing – review & editing. **Rina Maharani:** Methodology, Validation, Formal analysis, Investigation, Data curation, Writing – original draft, Writing – review & editing. **Dinar Ajeng Kristiyanti:** Investigation, Data curation. **Irmawati:** Investigation, Data curation. **Niki Prastomo:** Writing – review & editing, Supervision. **Tika Adilah M:** Visualization.

## Data Availability

Potato Leaf Disease Dataset in Uncontrolled Environment (Original data) (Mendeley Data).
